# Topology-optimized silicon-based dual-mode 4 × 4 electro-optic switch

**DOI:** 10.1515/nanoph-2022-0259

**Published:** 2022-11-04

**Authors:** Jiaqi Niu, Shanglin Yang, Ting Zhou, Hao Jia, Xin Fu, Zhizun Zhao, Zhen Li, Gaolu Zhang, Changhua Chen, Lin Yang

**Affiliations:** State Key Laboratory on Integrated Optoelectronics, Institute of Semiconductors, Chinese Academy of Sciences, P.O. Box 912, Beijing 100083, China; College of Materials Science and Opto-Electronic Technology, University of Chinese Academy of Sciences, Beijing 100049, China

**Keywords:** electro-optical switch, multimode optical switch, on-chip optical interconnect systems, silicon photonics

## Abstract

Silicon-based optical switch is one of the key components for on-chip optical interconnect systems, and mode division multiplexing technology has been employed to boost optical switches’ channel capacity. However, the majority of the proven multimode optical switches have a switching time in the microsecond range, which is insufficient for some applications. In this paper, we design and experimentally demonstrate a high-speed dual-mode 4 × 4 optical switch based on a mode-diversity scheme, composed of four pairs of mode multiplexers and de-multiplexers, and two optimized single-mode 4 × 4 optical switches. Fast switching is enabled based on the carrier dispersion effect. At the same time, we improve the performances of the optical switch by reducing the number of optical switch units used in the 4 × 4 Spanke–Beneš architecture. Its power consumptions are reduced by ∼17%. Its insertion losses are within 8.8 dB in the wavelength range of 1525–1565 nm in the both sates of “through” and “all-cross”, while the optical signal-to-noise ratios are larger than 12.8 dB. Also, 50 Gbps data transmission experiments verify the device’s data transmission functionality.

## 1 Introduction

With the demands on the information throughput dramatically increasing, optical interconnects have attracted widespread attentions as a means of breaking the communication bottleneck due to its broad bandwidth and low power consumption [1, 2]. To expand the channel capacity of the optical interconnects, wavelength division multiplexing (WDM) technology has been deeply studied [3–5]. But WDM systems always require several laser sources for different wavelength channels. Based on the orthogonal feature of the different spatial modes, mode division multiplexing (MDM) technology provides a new dimension to improve the transmission capacity [6, 7], which allows a single wavelength to carry multiple parallel data signals without interfering with each other. Silicon photonics is considered as a promising platform for establishing chip-level MDM networks, taking its advantages of low loss, high integration density, and compatibility with complementary metal-oxide-semiconductor fabrication [8]. Recently, many silicon-based devices in MDM community have been demonstrated, such as mode multiplexers/de-multiplexers [9, 10], multimode splitters [11, 12] and multimode crossings [13].

A fundamental component of optical interconnect system is the optical switch, which is used for optical link switching at the nodes [14]. In the past decades, silicon-based optical switches have achieved remarkable results and various scales of optical switches have been proposed one after another [15–19] from 4 × 4 to 64 × 64 and more. With the introduction of MDM, multimode optical switches were also proven [20–28], which commonly rely on Mach–Zehnder interferometer (MZI) structure or micro-ring structure. For compatibility with MDM systems, there exist two paths for multimode signal switching. One is to process multimode signals simultaneously by utilizing various mode-insensitive elements to compose optical switches. Based on a mode-insensitive phase shifter integrated into a balanced MZI structure, a three-mode 2 × 2 optical switch has been demonstrated in [25], while this kind of optical switch cannot achieve different routing functions separately for different spatial mode channels and exhibits difficulty in mode scalability. The other is to demultiplex the input multimode signals into fundamental modes and then use single-mode optical switches to handle each separately. In Refs. [26, 27], WDM-compatible MDM optical switches based on micro-ring structure are proposed. Although both the micro-ring optical switch and the Mach–Zehnder optical switch can achieve signal exchange, the latter is preferred because of its larger tolerance to fabrication errors and lower temperature sensitivity. However, most demonstrated multimode optical switches based on MZI structure belong to multimode 2 × 2 optical switches, and their large-scale application needs to be studied. Moreover, to the best of our knowledge, almost all the broadband multimode optical switches reported so far are based on thermo-optic (TO) tuning with the response time in the order of microseconds, which is not suitable for some application scenarios that require fast response [29].

In this paper, we design and experimentally demonstrate a topology-optimized silicon-based dual-mode 4 × 4 electro-optic (EO) switch. Based on the mode-diversity-processing idea mentioned above, the multimode optical switch divides the TE_0_ and TE_1_ modes into two groups of fundamental modes by mode demultiplexers according to the mode orders, and the two group of signals are processed separately by two single-mode 4 × 4 optical switches. In this procedure, the single-mode 4 × 4 optical switch is based on the optimized Spanke–Beneš architecture, which replaces one optical switch unit (OSU) with one waveguide crossing while maintaining the 4 × 4 reconfigurable non-blocking function. Since the waveguide crossing is passive and has lower insertion loss (IL) and optical crosstalk than the OSU, this operation reduces the overall power consumption and ILs, and thus improves the quality of the transmitted signal. Furthermore, The PIN modulation arms based on the carrier dispersion effect are embedded in the OSU, which enhances the response speed. The device’s footprint is 1 × 3 mm^2^. For this demonstration, the experimental results show that the measured ILs are less than 8.8 dB and the optical signal-to-noise ratios (OSNRs) are larger than 12.8 dB in the wavelength range of 1525–1565 nm. The switching time of the device is 4.4 ns. Also, data transmission experiments using 50 Gbps PAM4 optical signals indicate that the signals are not deteriorated significantly after passing through this device.

The remainder of this article is organized as follows. In Section 2, we describe the design of the dual-mode 4 × 4 optical switch. Section 3 presents the performances of the OSU and dual-mode 4 × 4 optical switch. Finally, in Section 4, we make a conclusion.

## 2 Design of the device

### 2.1 Optimized Spanke–Beneš optical switch

The input–output (I–O) mapping relationships of a 2 × 2 OSU can be completely described by the permutation matrix as follows,

(1) $$S_{\text{bar}}=\left(\begin{array}{cc} 1 & 0\\ 0 & 1 \end{array}\right),\;S_{\text{cross}}=\left(\begin{array}{cc} 0 & 1\\ 1 & 0 \end{array}\right),$$

where *S*_bar_ and *S*_cross_ correspond to the I–O mapping relationships of the OSU in the bar and cross states, respectively.

**Figure 1:**
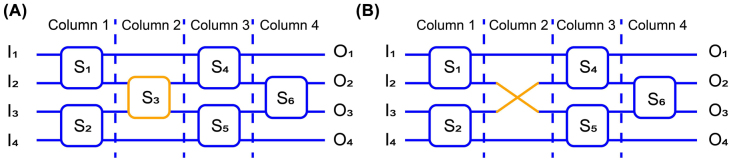
4 × 4 Spanke–Beneš optical switch. (A) Conventional architecture. (B) Improved architecture.

Reconfigurable non-blocking is an essential property to realize perfect switching functions. In order to achieve this feature, the routing states need to be able to realize all possible I–O mapping relationships. A 4 × 4 optical switch suggests that the number of routing states is 4! = 24, which implying that the number of different permutation matrices corresponding to its routing states is 24. As shown in Figure 1A, a 4 × 4 optical switch based on the Spanke-Beneš architecture can be divided into four columns. Thus, the I–O configurations can be written as follows,

(2) $$\left(\begin{array}{c} O_{1}\\ O_{2}\\ O_{3}\\ O_{4} \end{array}\right)=T_{\text{Switch}}\cdot \left(\begin{array}{c} I_{1}\\ I_{2}\\ I_{3}\\ I_{4} \end{array}\right),$$

(3) $$T_{\text{Switch}}=T_{4}\cdot T_{3}\cdot T_{2}\cdot T_{1},$$

where *T*_Switch_ is the permutation matrix of the 4 × 4 optical switch, and *T*_1_ ∼ *T*_4_ are the permutation matrices of Columns 1–4, respectively. Taking the matrices of the OSUs into Eq. (3), the permutation matrix of the 4 × 4 optical switch of the Spanke–Beneš architecture can be written as follows,

(4) $$\begin{array}{ll} T_{\text{Switch}} & =\left(\begin{array}{ccc} 1 & O_{1\times 2} & 0\\ O_{2\times 1} & S_{6}^{B|C} & O_{2\times 1}\\ 0 & O_{1\times 2} & 1 \end{array}\right)\cdot \left(\begin{array}{cc} S_{4}^{B|C} & O_{2\times 2}\\ O_{2\times 2} & S_{5}^{B|C} \end{array}\right)\\ & \;\cdot \left(\begin{array}{ccc} 1 & O_{1\times 2} & 0\\ O_{2\times 1} & S_{3}^{B|C} & O_{2\times 1}\\ 0 & O_{1\times 2} & 1 \end{array}\right)\cdot \left(\begin{array}{cc} S_{1}^{B|C} & O_{2\times 2}\\ O_{2\times 2} & S_{2}^{B|C} \end{array}\right), \end{array}$$

where $S_{1}^{B|C}$ ∼ $S_{6}^{B|C}$ corresponds to the transfer matrices of the OSUs from *S*_1_ to *S*_6_ in cross or bar state, respectively, which corresponding to the value of *S*_bar_ or *S*_cross_ in Eqs. (1) and (4) describes all the I–O relationships for the 4 × 4 optical switch by traversing the states of all OSUs. And it is easy to verify that the number of different matrices *T*_Switch_ is 24.

In the optimized 4 × 4 switch, as shown in Figure 1B, we use a waveguide crossing to replace the OSU *S*_3_ in Figure 1A. The permutation matrix of the new architecture based on four columns is:

(5) $$\begin{array}{ll} T_{\text{Switch}}^{\text{New}} & =\left(\begin{array}{ccc} 1 & O_{1\times 2} & 0\\ O_{2\times 1} & S_{6}^{B|C} & O_{2\times 1}\\ 0 & O_{1\times 2} & 1 \end{array}\right)\cdot \left(\begin{array}{cc} S_{4}^{B|C} & O_{2\times 2}\\ O_{2\times 2} & S_{5}^{B|C} \end{array}\right)\\ & \;\cdot \left(\begin{array}{cccc} 1 & 0 & 0 & 0\\ 0 & 0 & 1 & 0\\ 0 & 1 & 0 & 0\\ 0 & 0 & 0 & 1 \end{array}\right)\cdot \left(\begin{array}{cc} S_{1}^{B|C} & O_{2\times 2}\\ O_{2\times 2} & S_{2}^{B|C} \end{array}\right), \end{array}$$

where $T_{\text{Switch}}^{\text{New}}$ is the permutation matrix of the optimized 4 × 4 optical switch architecture. It can be verified that the number of different $T_{\text{Switch}}^{\text{New}}$ is still 24, meaning that the optimized architecture can realize the same I–O configuration function. Thus, the reconfigurable non-blocking nature of the Spanke–Beneš architecture is maintained. The routing complexity of the optical switch is reduced by replacing one OSU with one waveguide crossing. Meanwhile, considering that a waveguide crossing is better than OSU in terms of IL and optical crosstalk [30], the performances of the optical switch get improved. In addition, waveguide crossing typically has smaller size, facilitating higher integration density and hence lower cost.

### 2.2 Dual-mode 4 × 4 optical switch architecture

To introduce MDM in the EO switch, the multimode optical switch is constructed by the aforementioned mode-diversity processing. First, the multimode signals are demultiplexed into fundamental modes and bundled according to their mode orders. Single-mode 4 × 4 optical switches are then used to switch optical signals within each spatial mode separately, and finally, the output signals are combined by mode multiplexers.

**Figure 2:**
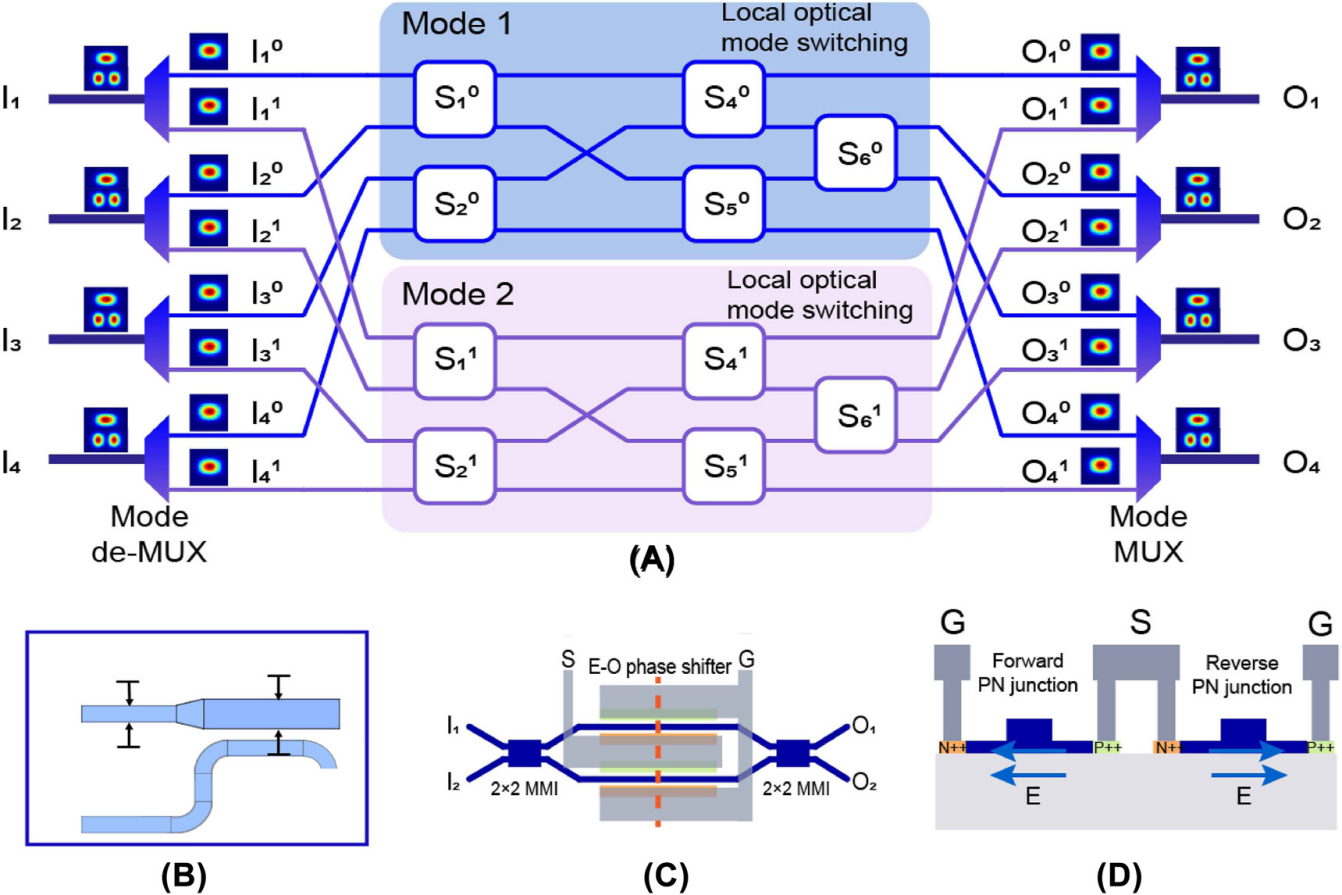
The dual-mode 4 × 4 optical switch. (A) Main schematic. (B) Mode multiplexer based on asymmetric directional coupler. (C) Signal-mode 2 × 2 EO switch unit with push–pull drive. (D) The cross section at the red line of (C).

The dual-mode 4 × 4 optical switch is schematically shown in Figure 2A. Four pairs of multimode waveguides constitute four pairs of multimode inputs and outputs, denoted as *I*_1_ to *I*_4_ and *O*_1_ to *O*_4_, respectively. The light from the modes TE_0_ and TE_1_ of the *i*th (*i* = 1–4) input port is converted to two fundamental mode signals denoted as *I*_i_^0^ and *I*_i_^1^ by the mode demultiplexer, respectively. The divided signals go into the corresponding *i*th input ports of the two identical single-mode 4 × 4 optical switches, respectively. Finally, the corresponding output signals *O*_*i*_^0^ and *O*_*i*_^1^ of the two single-mode 4 × 4 optical switches are connected to four dual-mode multiplexers to compose *O*_*i*_. For both modes, the routing function of the two single-mode 4 × 4 optical switch can be described by two permutation matrices, respectively, as follows,

(6) $$\begin{array}{ll} & \left(\begin{array}{cccc} O_{1}^{0} & O_{2}^{0} & O_{3}^{0} & O_{4}^{0} \end{array}\right)\\ & \;=T_{\text{Switch}}^{0}\cdot \left(\begin{array}{cccc} I_{1}^{0} & I_{2}^{0} & I_{2}^{0} & I_{2}^{0} \end{array}\right), \end{array}$$

(7) $$\begin{array}{ll} & \left(\begin{array}{cccc} O_{1}^{1} & O_{2}^{1} & O_{3}^{1} & O_{4}^{1} \end{array}\right)\\ & \;=T_{\text{Switch}}^{1}\cdot \left(\begin{array}{cccc} I_{1}^{1} & I_{2}^{1} & I_{2}^{1} & I_{2}^{1} \end{array}\right), \end{array}$$

where $T_{\text{Switch}}^{0}$ and $T_{\text{Switch}}^{1}$ , are the permutation matrices of the two single-mode 4 × 4 optical switches corresponding to the TE_0_ and TE_1_ modes, respectively. This multimode optical switch not only realize the signal exchange within the same spatial mode channels, at the same time realize the recombination between different spatial mode channels. Therefore, A total of 576 (4! × 4! = 576) routing states can be achieved by this dual-mode 4 × 4 optical switch.

In practical design, we use an asymmetric directional coupler (ADC) as a multiplexing/demultiplexing device for the TE_0_ and TE_1_ modes. As shown in Figure 2B, the widths of the ridge waveguides supporting the TE_0_ and TE_1_ modes are 0.40 μm and 0.92 μm, respectively, on a 220 nm high silicon rib waveguide with 70 nm in slab thickness. The ADC is the same as that reported in [31], whose IL is about 0.3 dB in the wavelength range of 1525–1565 nm.

As shown in Figure 2C, OSU is based on a 2 × 2 symmetric MZI structure with two equal-length modulation arms and two 3 dB power splitters, which are the multimode interference couplers (MMIs) with 6 μm in width and 43 μm in length. Each modulation arm contains a 200 μm-long lateral PIN diode, where the slab thickness and the rib width are 70 nm and 500 nm, respectively and highly doped P++ and N++ regions are defined 600 nm distant from the rib waveguide’s edge as shown in Figure 2D. A push-pull drive is integrated in the OSU. Both arms can be modulated respectively with only one set of push-pull electrode. The polarity of the voltage on electrode S determines which arm is modulated. When the arms’ PIN diode is in the forward bias state, free carriers will be injected into it and induces a decreased modulation of the effective index of the arms. Compared with single arm modulation, this kind of drive helps decrease the optical loss caused by many injected carriers, and alleviate the impacts of thermal effect due to excessive voltage on PIN in some extent [32].

### 2.3 Device fabrication

**Figure 3:**
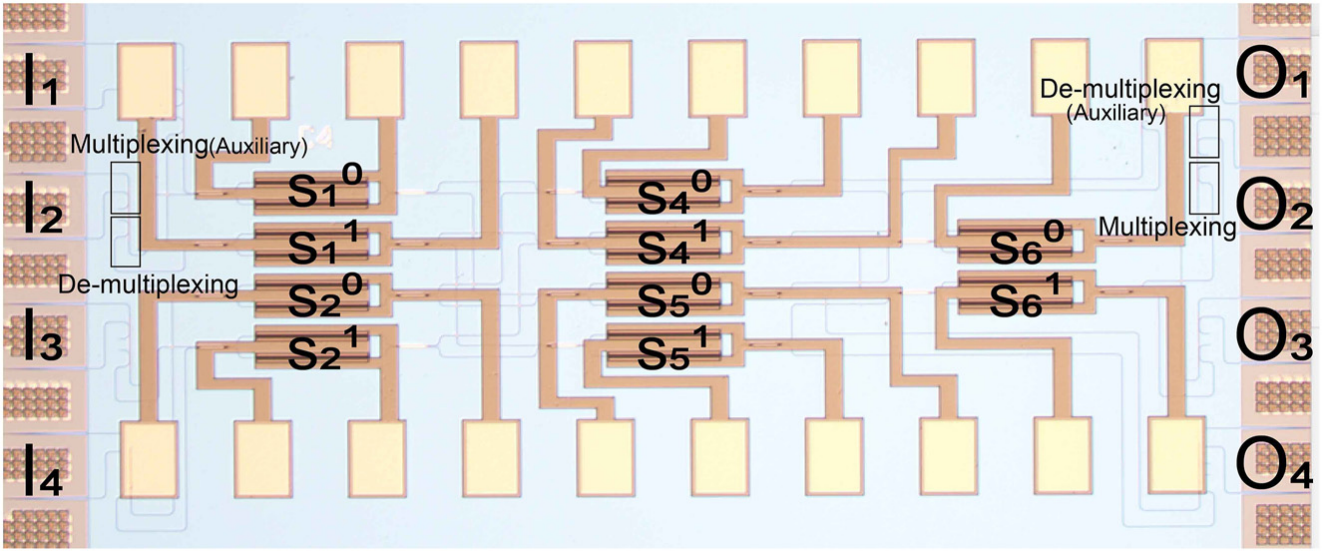
Optical microscope image of the fabricated dual-mode 4 × 4 electro-optical switch.

The optimized dual-mode 4 × 4 optical switch, with a footprint of 1 × 3 mm^2^, is fabricated on an 8-inch SOI wafer with a 220 nm thick top silicon layer and a 3 μm thick buried silicon dioxide layer by the CMOS compatible process at Advanced Micro Foundry, Singapore. Since the coupling structures between single-mode fibers and devices are commonly used for signal input and output in experiments, four pairs of auxiliary mode multiplexers and de-multiplexers are integrated in the device. The micrograph of the fabricated device is shown in Figure 3.

## 3 Experiment and result analysis

### 3.1 2 × 2 electro-optic switch unit

**Figure 4:**
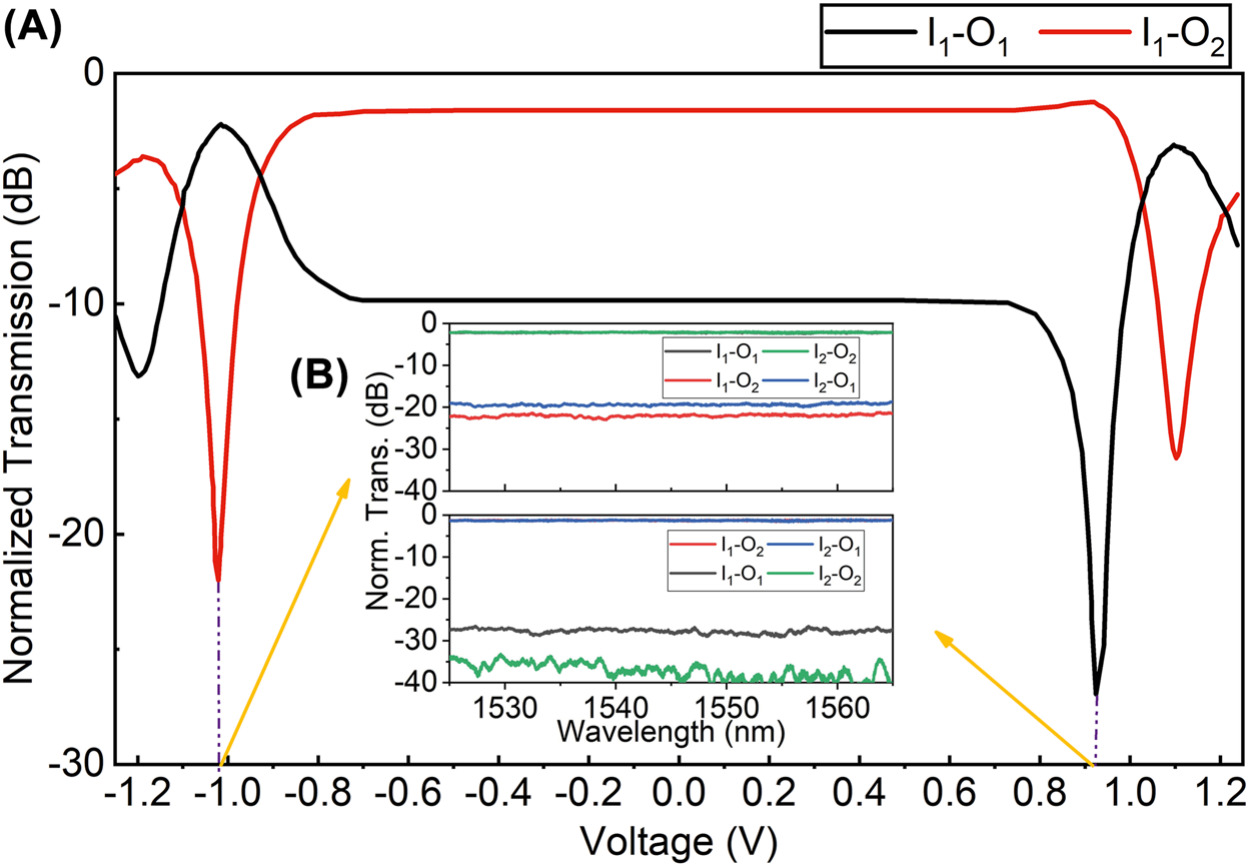
The performance of the OSU. (A) Measured output power of the OSU vs. applied voltage on the electrode S. (B) The normalized transmission spectra for both states.

First, we measured the switching performance of the OSU shown in Figure 2C. Figure 4A presents the cross and bar states are achieved respectively when the operating voltages of the tested OSU are 0.92 V and −1.02 V. Based on these results, Figure 4B shows the static transmission spectra of the OSU in the bar and cross states in the wavelength range of 1525–1565 nm. In the cross state, the optical crosstalk is less than −26 dB. However, in the bar state the optical crosstalk is deteriorated to −17 dB due to the imbalance between the optical powers of the two arms caused by the free carrier absorption, which simultaneously makes the optical IL be increased by 0.7–1.1 dB compared with the cross state. In addition, it is obvious that in the cross state the crosstalk for the two links of *I*_1_–*O*_1_ and *I*_2_–*O*_2_ is different. Similarly, in the bar state the crosstalk for the two links of *I*_1_–*O*_2_ and *I*_2_–*O*_1_ is also different. This problem may be caused by the imperfections of the MMIs and can be resolved by improving the fabrication.

### 3.2 Transmission spectra of the optimized dual-mode 4 × 4 optical switch

**Figure 5:**
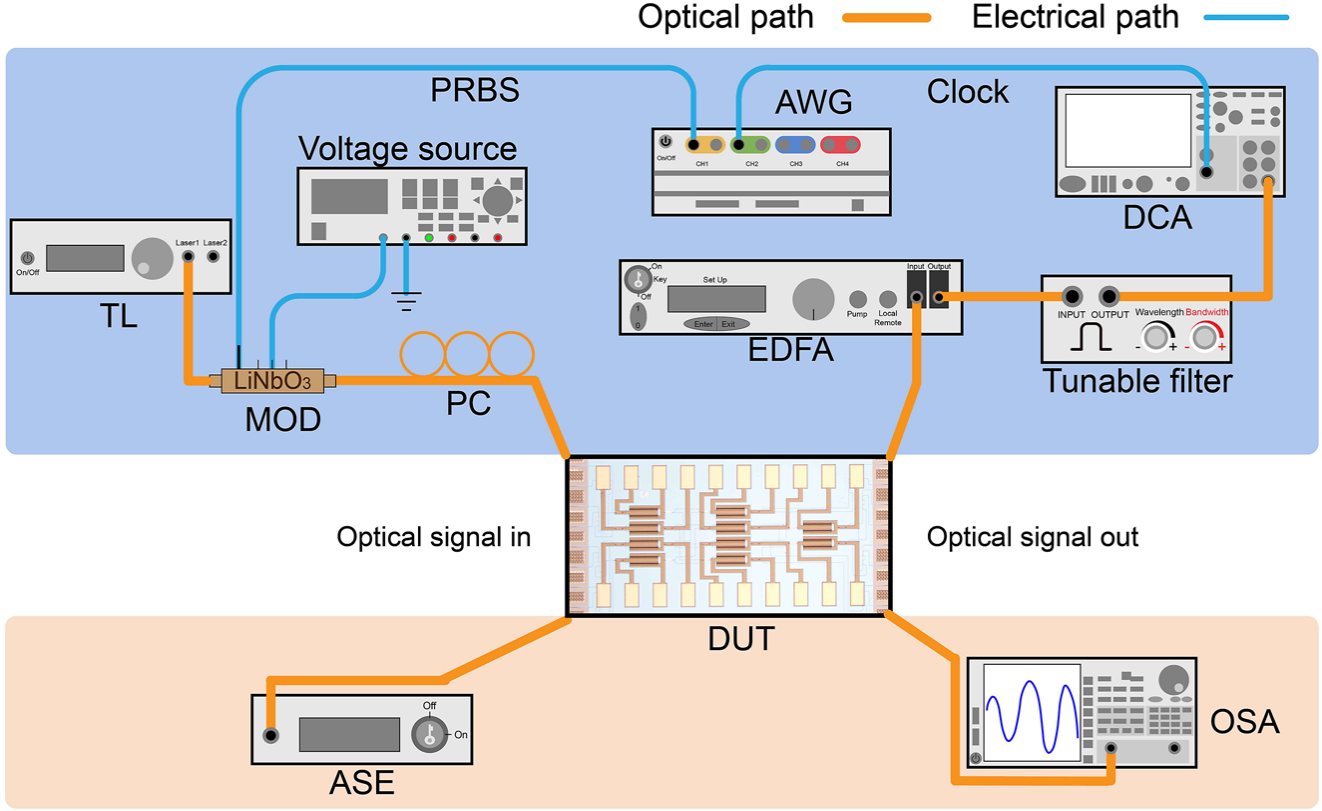
Experimental setup for characterizing the device (TL, tunable laser; ASE, amplified spontaneous emission; MOD, modulator; PC, polarization controller; AWG, arbitrary waveform generator; DUT, device under test; DCA, digital communication analyzer; OSA, optical spectrum analyzer).

In the lower half of Figure 5, the experimental setup for characterizing the transmission spectra of the device is displayed. The broad-spectrum light generated from the amplified spontaneous emission source is coupled into the device through an optical fiber with the spot size of 5 μm, and the output light is fed into an optical spectrum analyzer for spectrum measurement. By controlling the driving voltages applied to the phase shifters of the OSUs, the dual-mode 4 × 4 optical switch can be configured in any of the 576 routing states. Here we chose the “through” and “all-cross” states as examples to verify the device’s transmission characterizations. The established links and states of 10 OSUs for the corresponding routing states are listed in Table 1.

**Table 1:** The switch’s states and the corresponding optical links.

State of the switch	Optical links					States of OSUs				
“Through”	TE_0_	I_1_^0^ → O_1_^0^	I_2_^0^ → O_2_^0^	I_3_^0^ → O_3_^0^	I_4_^0^ → O_4_^0^	S_1_^0^	S_2_^0^	S_4_^0^	S_5_^0^	S_6_^0^
						B	B	B	B	C
	TE_1_	I_1_^1^ → O_1_^1^	I_2_^1^ → O_2_^1^	I_3_^1^ → O_3_^1^	I_4_^1^ → O_4_^1^	S_1_^1^	S_2_^1^	S_4_^1^	S_5_^1^	S_6_^1^
						B	B	B	B	C
“All-cross”	TE_0_	I_1_^0^ → O_4_^0^	I_2_^0^ → O_3_^0^	I_3_^0^ → O_2_^0^	I_4_^0^ → O_1_^0^	S_1_^0^	S_2_^0^	S_4_^0^	S_5_^0^	S_6_^0^
						C	C	C	C	C
	E_1_	I_1_^1^ → O_4_^1^	I_2_^1^ → O_3_^1^	I_3_^1^ → O_2_^1^	I_4_^1^ → O_1_^1^	S_1_^1^	S_2_^1^	S_4_^1^	S_5_^1^	S_6_^1^
						C	C	C	C	C

**Figure 6:**
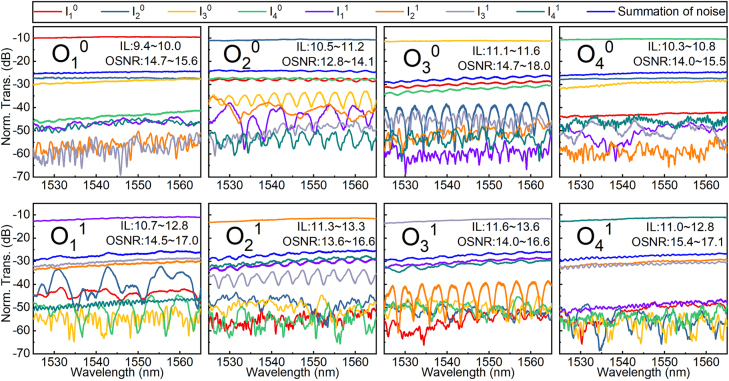
Normalized transmission spectra of the dual-mode 4 × 4 optical switch in the “through” state.

The measured spectra of the optical switch in the “through” state is shown in Figure 6. Each plot includes eight transmission spectra from all eight input ports to one output port and the worst noise spectra for the optical link, which are the summation of the noises from all input ports except the one which signals come from. As illustrated in Figure 6, in the wavelength range of 1525–1565 nm, the ILs of the optical switch chip are within 9.4–13.6 dB in the “through” state. The coupling loss between the device and the lensed fiber is estimated by measuring a reference straight waveguide with the same couplers. It is about 2.4 ± 0.2 dB/butt. Therefore, the on-chip ILs of the dual-mode 4 × 4 optical switch are less than 8.8 dB in the wavelength range of 1525–1565 nm. Here, the on-chip ILs of the dual-mode optical switch are composed of four parts: ILs of the waveguide crossings (about 0.05 dB per junction), ILs of the OSUs, ILs of the connected waveguides (about 2.5 dB/cm) and ILs of the mode multiplexers and de-multiplexers (0.1–0.4 dB per junction). The worst noise spectra show the OSNRs are all larger than 12.8 dB over 40 nm for the two modes. Theoretically, the optical noise comes from the optical inter-mode crosstalk and the optical inter-path crosstalk, while the noise transmission spectra from the other spatial mode channels lie beneath every plot. Thus, the optical inter-mode crosstalk from ADCs can be largely negligible compared to the optical crosstalk from other aspects. The loss imbalance in two arms of the MZI OSUs is the main reason for the poor crosstalk. OSUs with typical structures can help get better crosstalk. For example, a dilated 2 × 2 MZI was used in an EO switch unit to achieve an optical crosstalk of −31 dB in [33]. In Ref. [34], the optical crosstalk of −50 dB in an OSU is realized by using a variable splitter to substitute the front 3 dB coupler of MZI.

**Figure 7:**
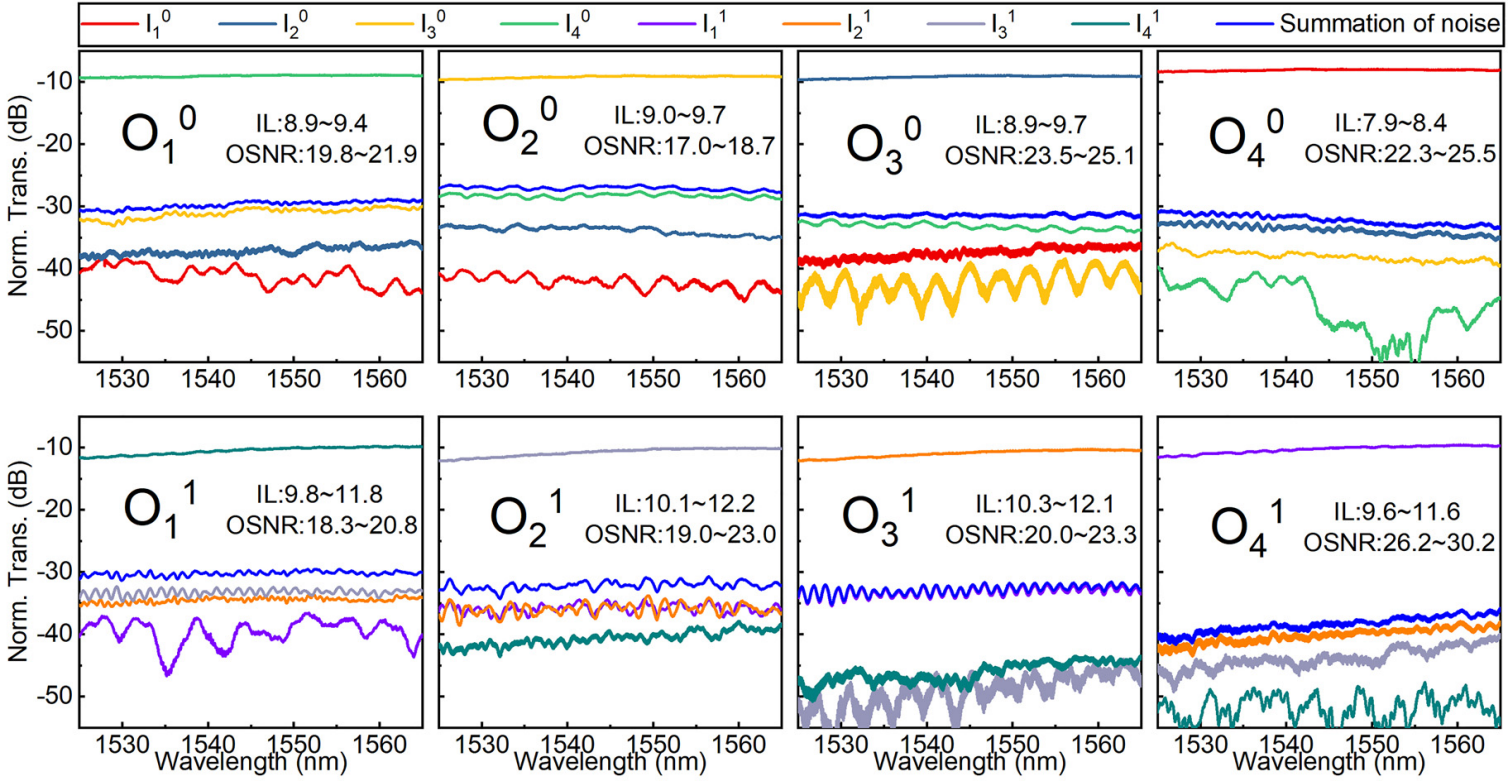
Normalized transmission spectra of the dual-mode 4 × 4 optical switch in the “all-cross” state.

We then toggled the dual-mode 4 × 4 optical switch to the “all-cross” state by tuning the applied voltages on the phase shifters of OSUs. Because optical inter-mode crosstalk is nearly negligible, we simply characterized the transmission spectra of the dual-mode optical switch within each group of spatial mode channels in the “all-cross” state. As shown in Figure 7, each plot only includes four transmission spectra within the same spatial mode channels and the worst noise spectrum. Due to the dispersion of the ADCs and OSUs, the transmission spectra of the TE_1_ channels present slightly wavelength-dependent. Its on-chip ILs are within 7.4 dB (12.2–2.4 × 2 = 7.4) and the OSNRs are larger than 17.0 dB in the wavelength range of 1525–1565 nm. There is much difference between the two routing states in terms of ILs and OSNRs. In the future, an optical phase-bias structure can be brought into the MZI phase-shifted arm to remove the difference between the cross and bar states of OSU and enable the dual-mode optical switch to have a relatively balanced performance in various states [35].

### 3.3 Data transmission

**Figure 8:**
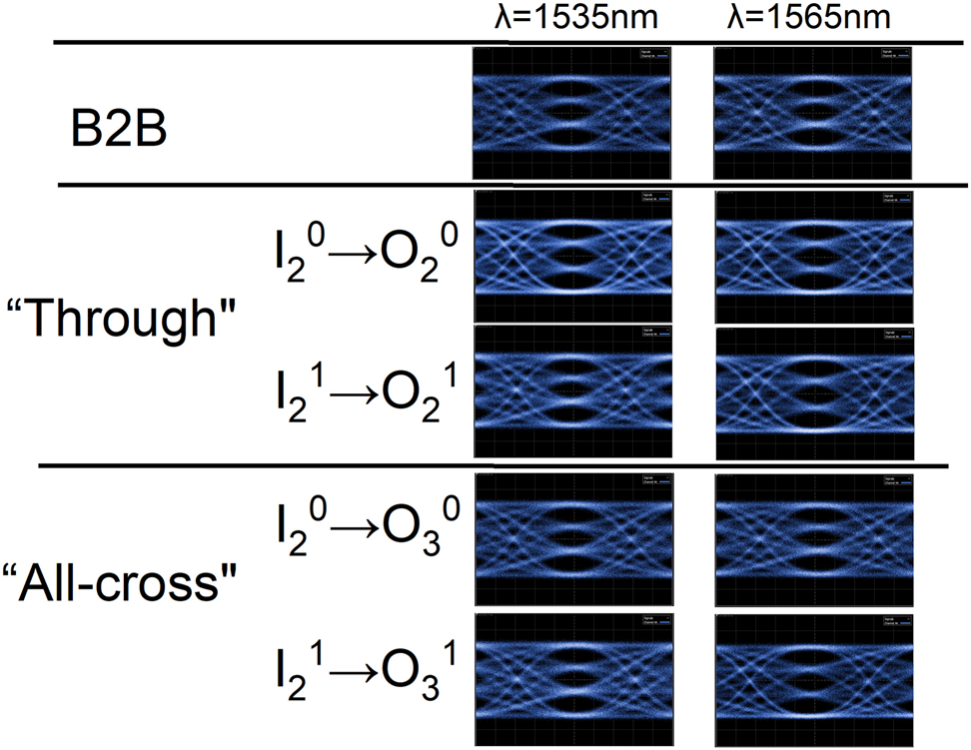
Eye diagrams for 50 Gb/s data transmission at 1535  and 1565 nm, respectively.

The experimental setup characterizing the data transmission is shown in the upper half of Figure 5. Continuous-wave light is emitted by a tunable laser and modulated by a LiNbO_3_ modulator, which is driven by 50 Gbps PAM signals with a length of 2^31^ − 1. After being adjusted to transverse electric polarization by a polarization controller, the light enters a straight waveguide on the chip. Then, amplified by an erbium-doped fiber amplifier and filtered by a tunable filter, the optical signals are sent to the digital communication analyzer (DCA) to observe the back-to-back (B2B) eye diagram. Next, the straight waveguide is substituted by the device under test (DUT) for observing the data transmission of the device. In the “through” and “all-bar” states, the optical links with input ports of *I*_2_^*m*^ (*m* = 0 or 1) pass through the most OSUs. These links are essentially the worst-case scenarios for all links in the two states. 50 Gbps data transmission experiments are implemented for these optical links in the “through” and “all-cross” states, at 1535 nm and 1565 nm, respectively. As shown in Figure 8, compared with the B2B eye diagram, the eye diagrams of the optical links show slight deterioration, but still keeps open and clear. It is caused by the ILs of the measured optical links. Therefore, it illustrates that the device is capable of switching 50 Gbps PAM4 optical signals with high signal integrity for the dual modes, even though it has a relatively high ILs.

### 3.4 The time-domain response and power consumption

**Figure 9:**
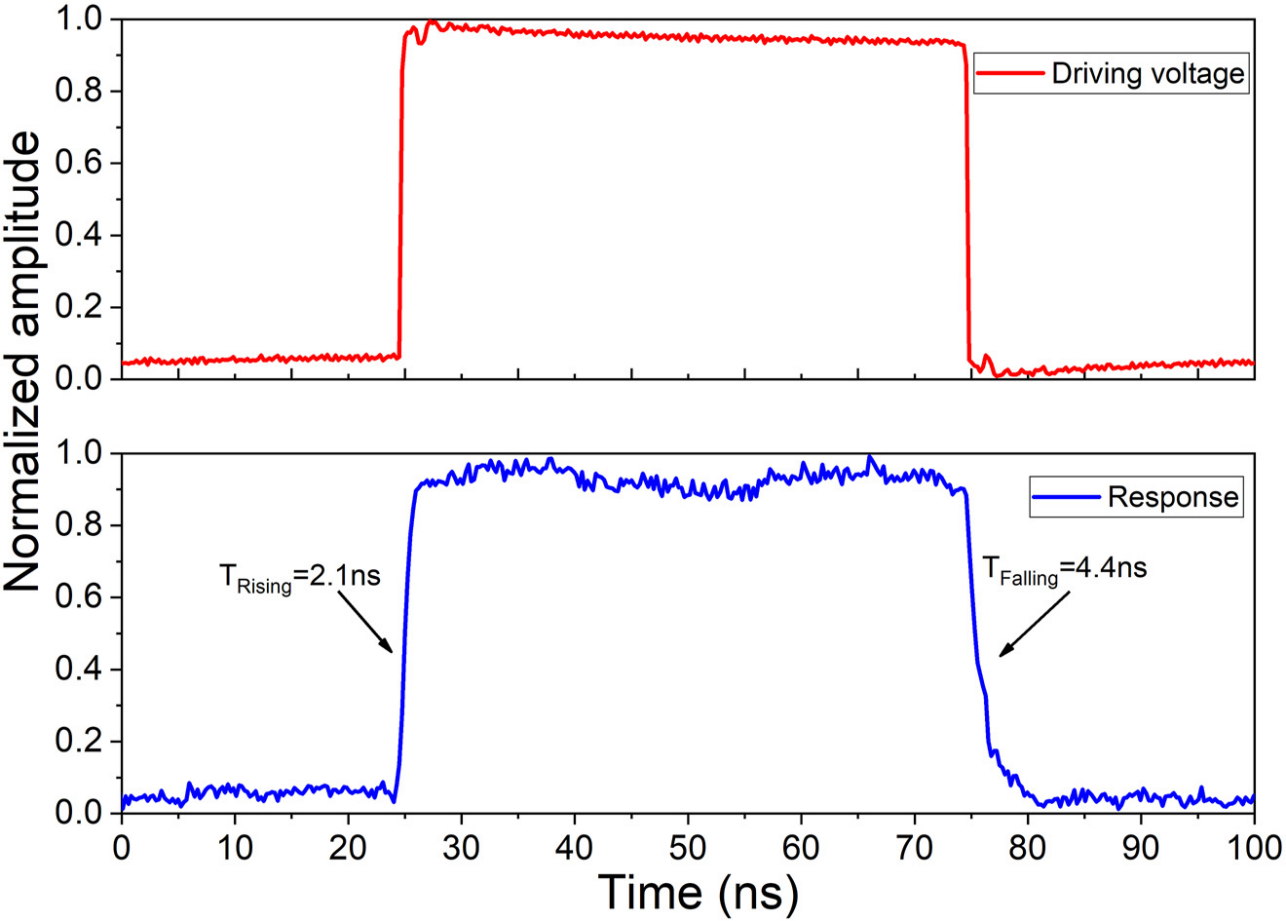
The driving signal and dynamic response of the OSU.

By measuring the time-domain response of the OSU, we characterized the switching speed of the device. The states of the OSU are toggled by applying a 10 MHz square wave electrical signals to the S pad, and a DCA is used to record the dynamic response time of the output signals. The device’s response speed is decided by the OSU, whose 10–90% rising and falling times are 2.1 ns and 4.4 ns, respectively, as illustrated in Figure 9. In addition, the power consumption of each OSU is measured, as listed in Table 2. The minimum and maximum power consumptions of the dual-mode 4 × 4 optical switch are 9.2 mW and 52.1 mW, respectively. The power consumptions can be reduced in the future by extending the length of PINs in the modulation arms.

**Table 2:** Driving voltages and power consumptions of all the optical switch units (OSUs) under the cross and bar states.

	OSU		S_1_^0^	S_2_^0^	S_4_^0^	S_5_^0^	S_6_^0^
Switch array 1	Cross	Voltage (V)	−0.89	−0.90	−0.94	−0.80	0.92
		Current (mA)	−1	−1	−2	0	1
		Power (mW)	0.89	0.90	1.88	0	0.92
	Bar	Voltage (V)	1.01	1.00	0.99	1.03	−1.01
		Current (mA)	6	5	5	7	−5
		Power (mW)	6.06	5.00	4.95	7.21	5.05

	OSU		S_1_^1^	S_2_^1^	S_4_^1^	S_5_^1^	S_6_^1^
Switch array 2	Cross	Voltage (V)	0.93	0.92	0.93	0.92	0.91
		Current (mA)	1	1	1	1	1
		Power (mW)	0.93	0.92	0.93	0.92	0.91
	Bar	Voltage (V)	−0.99	−0.99	−1.00	−0.99	−1.00
		Current (mA)	−4	−5	−5	−5	−5
		Power (mW)	3.96	4.95	5.00	4.95	5.00

## 4 Conclusions

In conclusion, a dual-mode 4 × 4 EO switch based on an optimized Spanke–Beneš architecture is demonstrated, whose 10–90% rising and falling time of the device are about 2.1 ns and 4.4 ns. At the same time, the device’s performances get improved through topology optimization and are characterized in the “through” and “all-cross” states respectively. The ILs are less than 8.8 dB and its measured OSNRs are larger than 12.8 dB in both states, in the wavelength range of 1525–1565 nm. In addition, 50 Gbps data transmission experiments are implemented to verify its transmission functionality. Overall, the demonstrated high-speed dual-mode 4 × 4 silicon optical switch shows promising potential for future large-capacity optical MDM networks. Furthermore, the design ideas, which combines a mode-diversity scheme and a topological optimization method, may enlighten the researches of other optical interconnect devices.
